# *Mentha longifolia* assisted nanostructures: An approach to obliterate microbial biofilms

**DOI:** 10.1371/journal.pone.0303521

**Published:** 2024-07-10

**Authors:** Mahwish Batool Kazmi, Hayfa Habes Almutairi, Ayesha Andleeb, Raheela Jabeen, Ghulam Mustafa, Umm-e- Habiba, Safdar Abbas Kazmi, Farah Naz, Najma Qammar

**Affiliations:** 1 Department of Biochemistry & Biotechnology, The Women University Multan, Punjab, Pakistan; 2 Department of Chemistry, College of Science, King Faisal University, Al-Ahsa, Saudi Arabia; 3 Department of Biochemistry, Government College University Faisalabad, Faisalabad, Punjab, Pakistan; 4 Department of Environmental Science, COMSATS, Abbottabad Campus, Abbottabad, Pakistan; 5 Department of Statistics, The Women University Multan, Punjab, Pakistan; 6 Department of Biochemistry, Bahauddin Zakariya University, Multan, Pakistan; VIT University, INDIA

## Abstract

Microbes maneuver strategies to become incessant and biofilms perfectly play a role in scaling up virulence to cause long-lasting infections. The present study was designed to assess the use of an eco-friendly formulation of functionalized silver nanoparticles generated from *Mentha longifolia* leaf extract (MℓE) for the treatment of biofilm-producing microbes. Nanoparticles synthesized using MℓE as a reducing agent were optimized at different strengths of AgNO_3_ (1 mM, 2 mM, 3 mM, and 4 mM). Synthesis *of M*. *longifolia* silver nanoparticles (MℓAgNPs) was observed spectrophotometrically (450 nm) showing that MℓAgNPs (4 mM) had the highest absorbance. Various techniques e.g., Fourier transforms Infrared spectroscopy (FTIR), Dynamic light scattering (DLS), zeta potential (ZP), X-ray Diffraction (XRD), scanning electron microscope (SEM), and transmission electron microscope (TEM) were used to characterize MℓAgNPs. In the present study, the Kirby—Bauer method revealed 4mM was the most detrimental conc. of MℓAgNPs with MIC and MBC values of 0.62 μg/mL and 1.25 μg/mL, 0.03 μg/mL and 0.078 μg/mL, and 0.07 μg/mL and 0.15 μg/mL against previously isolated and identified clinical strains of *Escherichia coli*, *Pseudomonas aeruginosa*, *Klebsiella pneumoniae*, and *Staphylococcus aureus*, respectively. Moreover, the MℓAgNP antibiofilm activity was examined via tissue culture plate (TCP) assay that revealed biofilm inhibition of up to 87.09%, 85.6%, 83.11%, and 75.09% against *E*. *coli*, *P*. *aeruginosa*, *K*. *pneumonia*, and *S*. *aureus*, respectively. Herbal synthesized silver nanoparticles (MℓAgNPs) tend to have excellent antibacterial and antibiofilm properties and are promising for other biomedical applications involving the extrication of irksome biofilms. For our best knowledge, it is the first study on the use of the green-synthesized silver nanoparticle MℓAgNP as an antibiofilm agent, suggesting that this material has antibiotic, therapeutic, and industrial applications.

## Introduction

A biofilm is a community of microbial cells attached to a substrate and coated with a polysaccharide matrix that is highly resistant to host responses [1]. Biofilms can be established on a variety of substrates, such as on surviving tissues, essential medical devices, natural marine ecosystems, and industrial water systems. Medical devices play a significant role in providing inert surfaces for biofilm formation. Some examples include cardiac pacemakers, catheters, coronary stents, breast implants, contact lenses, dental implants, and prostheses, which are responsible for creating major public health problems [2, 3]. The biofilm formation process is a remarkable virulence factor in many other localized long-lasting infections [4]. Recently, many infections caused by antibiotic-resistant bacteria have become more common. Wounds, cholelithiasis, urinary tract infection (UTI), and nosocomial infections are common examples of microbial biofilms that are composed of interconnected, surface-attached bacterial populations that bind to living and nonliving substrates [1].

Nanotechnology is about to bring new incredible progress in the biomedical and clinical fields [5]. Advances in nanotechnology will provide a great tool in this area. It is stated that silver nanoparticles can move inside the biofilm. The penetration of nanoparticles (NPs) kills the bacteria in the lower layer of the biofilm. Silver nanoparticles are commonly used in biotechnology because of their distinct characteristics, such as conductivity, stabilization, catalytic, and bactericidal properties [6]. Recently, silver nanoparticles have been shown to be effective at removing bacterial biofilms. Silver nanoparticles are very important because of their distinctive physical and chemical characteristics, which include bactericidal, antibiofilm, and antioxidant properties [7]. Green synthesized silver nanoparticles are more advantageous than other nanoparticles because of their lower cost, greater speed, ecofriendly and time saving properties [8–10].

Currently, the use of biological agents to synthesize nanoparticles is becoming increasingly favorable as an alternative to traditional processes [11]. The green synthesis of nanoparticles is an interesting topic, and many biological processes are being applied for this purpose [12]. Utilizing plants in nanoparticle synthesis is an environmentally friendly approach because it avoids the use of harmful and polluting materials [13, 14]. The synthesis of nanoparticles is based on the reduction and neutralization of metal ions in solution [12, 15]. Medicinal plants are used as antiseptics, antivirals, antioxidants, or antibacterial compounds since they have no chemical toxic effects and have protective effects against various diseases and bacteria [16, 17]. Different plant extracts have been used in gold and silver nanoparticle synthesis in numerous studies. According to research conducted by Rao *et al*., [18], the key advantage of using plants for the synthesis of nanoparticles is that they are biosafe, simple to use, and contain a diverse range of metabolites involved in the ion reduction procedure. Nanoparticle synthesis via physical and chemical synthetic approaches is extremely expensive, but this cost can be significantly reduced by using natural herbs in the synthesis process [19].

*Mentha longifolia*, also known as horsemint, belongs to the Lamiaceae family and is native to South Africa, Asia, and Europe. It is an aromatic perennial herb with a high growth rate, a 40–120 cm tall stem and a creeping rhizome. It is utilized as a natural herbal remedy. The entire plant is utilized in medication, and the plant rhizome is often used as well [8]. Furthermore, the plant species have a wide range of uses in food, cosmetics, and medicine and have antimicrobial, antispasmodic, antiseptic, and expectoric effects also have effects on sore throat, cold symptoms, dyspepsia, and retching [8, 20]. *M*. *longifolia* leaves are widely accessible for the green synthesis of nanoparticles, as these plants produce a variety of natural terpenoids known as menthol that can be found in their essential oils [8]. The reducing and stabilizing ability of silver nanoparticles are linked to the flavonoids, terpenoids, and alkaloids found in the mint extract [21]. *M*. *longifolia* has excellent antibacterial properties and has been thoroughly examined against a variety of infectious and noninfectious bacterial species [22]. Due to its antibacterial properties, it is able to disrupt or inhibit biofilm formation. The aim of this research was to synthesize silver nanoparticles from *M*. *longifolia* leaf extract, characterize them by various techniques, evaluate their antibacterial activity and treat biofilms using silver nanoparticles.

## Materials and methods

### Preparation of *Mentha longifolia* leaf extract

**Fig 1:**
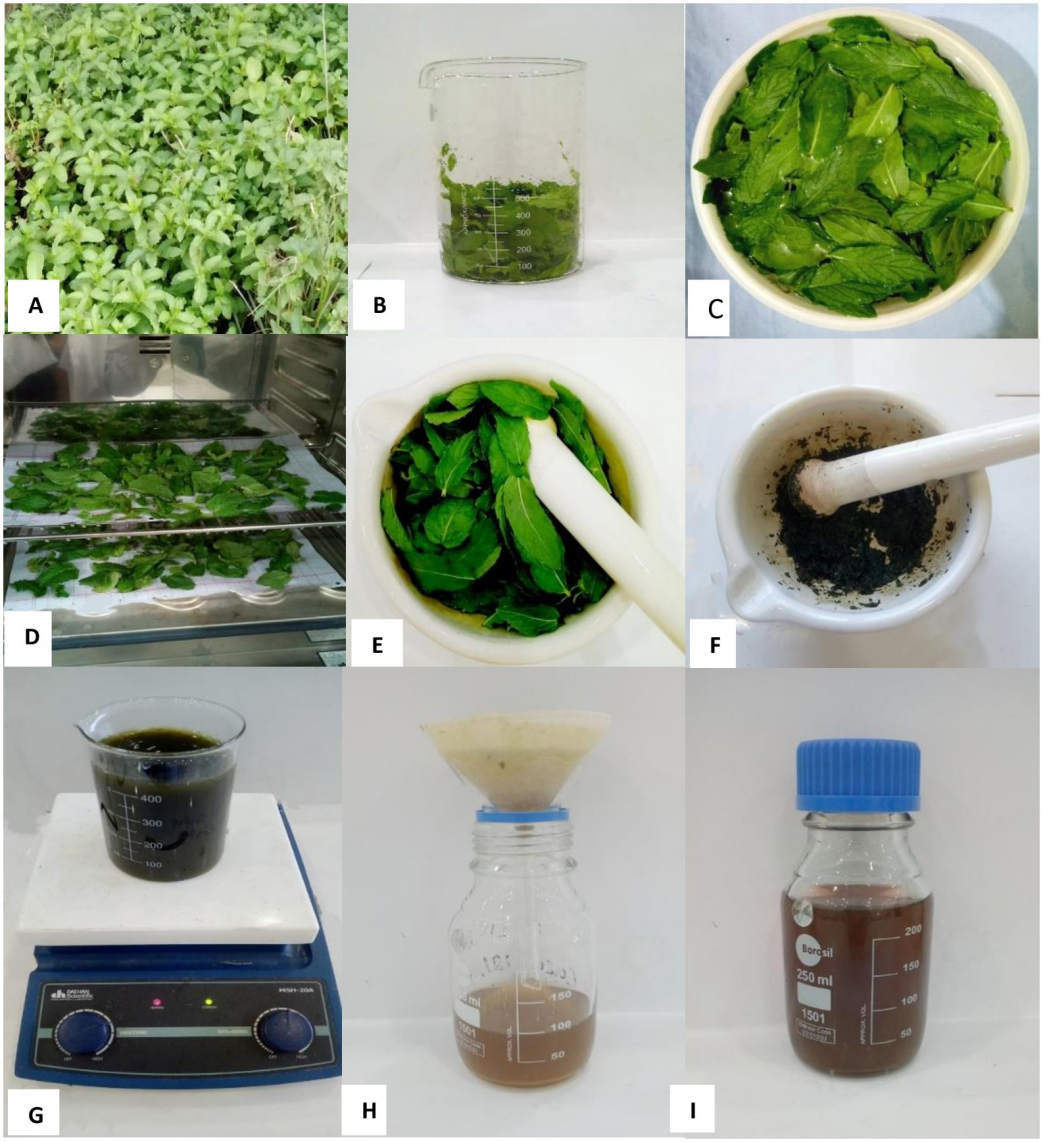
Preparation of plant extract. (A) *Mentha longifolia* plant. (B) *Mentha longifolia* leaves. (C) Washing of *M*. *longifolia* leaves. (D) Drying of *M*. *longifolia* leaves. (E) Pulverized leaves. (F) Pulverization of *M*. *longifolia* leaves. (G) Boiling and stirring content. (H) Content filtration. (I) Plant extract.

Fresh *M*. *longifolia* bushes were purchased from the local market of Multan and taxonomically verified from the Department of Botany, The Women University, Multan. Leaves of *M*. *longifolia* were separated from the stems and washed with distilled water. After washing, the *M*. *longifolia* leaves were kept on drying for one hour in a drying oven at 45°C [20]. Dried *M*. *longifolia* leaves were pulverized in a mortar and pestle, 5 g of pulverized leaves was mixed in 100 mL of sterilized distilled water, and the contents were boiled for 30 min. After boiling, the contents were filtered with Whatman No. 1 filter paper. A dark yellow extract was obtained and stored at 4°C for the synthesis of silver nanoparticles, as shown in Fig 1. The solutions were made in sterilized distilled water [23].

### Biosynthesis of silver nanoparticles using *Mentha longifolia*

**Fig 2:**
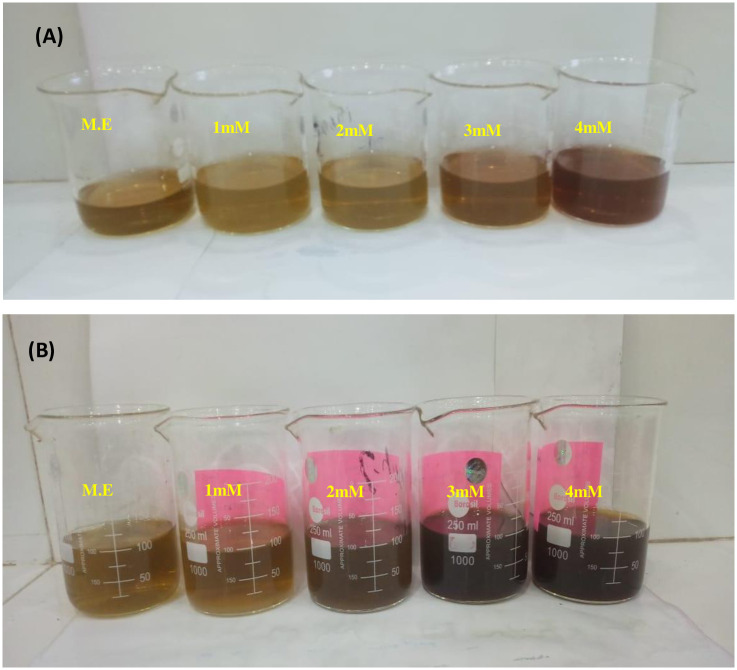
Visual inspection of silver nanoparticles. (M.E) *M*. *longifolia* leaf extract and reaction mixtures of different concentrations were mixed with 9:1 ratio (A) Reaction mixture of different concentrations at the time of preparation. (B) Reaction mixture after 24 hours.

For the synthesis of silver nanoparticles from *M*. *longifolia* leaf extract, 99% pure silver nitrate (Daejung, Korea) was utilized. To standardize the concentrations of silver nitrate solution, silver nanoparticles were synthesized at different concentrations (i.e., 1 mM, 2 mM, 3 mM, and 4 mM silver nitrate solution) [23]. The concentrations were chosen based on a literature survey, optimized by initial color change and further confirmed spectrophotometrically. MℓAgNPs were synthesized by reacting different concentrations (i.e., 1–4 mM) of silver nitrate solution with *M*. *longifolia* leaf extract at a 9:1 ratio and incubating at room temperature for 24 hours in the dark. Visual inspection of the reaction mixtures was performed at the time of preparation and after 24 hours, as shown in Fig 2. The color change resulting from the addition of different concentrations of silver nanoparticles indicated the synthesis of MℓAgNPs. NPs were separated by centrifugation by washing, drying in a drying oven, and storing at 4°C for further physical and biological analyses.

### Characterization of MℓAgNPs

The MℓAgNPs were characterized by visual inspection, UV‒Vis spectrophotometry, fourier transform infrared (FTIR), dynamic light scattering (DLS), zeta potential (ZP), scanning electron microscopy (SEM), transmission electron microscopy (TEM), and X-ray diffraction (XRD). Visual inspection is the first step, which shows the color variations of reaction mixtures. Spectral studies were conducted with a Dual Beam spectrophotometer (C-7200S, PEAK Instruments Inc., USA) at wavelengths ranging between 200 and 800 nm using glass cuvette cells. FTIR analysis was performed on a Bruker tensor 27 (USA) with Opus software with a range of 4000–400 cm^-1^. DLS was used to determine the size of the MℓAgNPs in solution. The surface charge on the silver nanoparticles was analyzed using a temperature-controlled Zetasizer Nano-ZS (Malvern, UK) by adjusting the temperature at 25°C at the 2 mm position. The morphology of the nanoparticles was studied using TEM (JEM-1010, JEOL, Japan). The crystalline structure of the silver nanoparticles was determined via XRD (JD-3532, JEOL, Japan). The surface morphology of the silver nanoparticles was analyzed via SEM [14].

### Determination of the antibacterial activity of silver nanoparticles by a well diffusion assay

The antibacterial activity of MℓAgNPs was determined by an agar well diffusion assay against our previously isolated and identified [24] four different clinical isolates, (Gram negative i.e., *Escherichia coli*, *Klebsiella pneumoniae*, *Pseudomonas aeruginosa*, and Gram positive i.e., *Staphylococcus aureus*) obtained from Tertiary Care Hospital, Multan., and identified qualitatively using CHROMagar medium as reported by Sahal *et al*., [25]. Four different concentrations of silver nanoparticles were used. Pathogenic cultures were grown in nutrient broth with constant shaking at 37°C for 24 hours for use in the antibacterial assay. Muller Hinton agar plates were prepared and swabbed with 24-hour-old broth culture of four different bacterial strains. Six wells were made on MHA plates with sterilized cork borer. A total of 100 μL of MℓAgNPs of four different strengths (i.e., 1–4 mM), resuspended in water, were added to four respective wells, and two wells were filled with mint extract and silver nitrate solution separately as control samples. The plates were subsequently placed in an incubator at 37°C for 24 hours, after which the inhibition zones were measured in millimeters [23].

### Determination of the minimum inhibitory concentration (MIC)

The broth dilution method was used to determine the MIC of MℓAgNPs at four different concentrations against four different types of bacteria. For this purpose, 96-well microtiter plates were used. Twofold serial dilutions of MℓAgNPs were used to determine 50% inhibition of microbial growth (IC_50_) by adjusting the concentrations of the agents from 10 μg/mL to 0.019 μg/mL. The bacterial suspensions were adjusted to 10^5^ CFU/mL in tryptic soy broth. The negative control contained broth with nanoparticles, and the positive control contained broth with bacteria. The plates were incubated for 24 hours at 37°C. After 24 hours, turbidity in each well was visually observed, and the results for each plate were noted. All the experiments were run in triplicate, and the means ± standard deviations were calculated [26].

### Determination of minimum bactericidal concentration (MBC)

After the MIC of the silver nanoparticles against the bacterial pathogens was determined, 50 μL of the suspension was taken from each well, which indicated no growth of the bacteria, and the mixture was inoculated on nutrient agar plates. The plates were incubated at 37°C for 24 hours, after which the plates were observed. The minimum inhibitory concentration at which there is no visible bacterial growth or inhibition of growth up to 99.5% is known as the minimum bactericidal concentration. This was done by examining the bacterial growth before and after incubating on nutrient agar plates [26].

### Antibiofilm effect of silver nanoparticles

The tissue culture plate method (TCP) was used to evaluate the antibiofilm activity of different concentrations of MℓAgNPs against four types of bacterial strains by a crystal violet assay [27]. An antibiofilm assay was performed to examine clinical isolates of *E*. *coli*, *Klebsiella pneumoniae*, *Pseudomonas aeruginosa* and *Staphylococcus aureus*. Briefly, a 96-well plate (flat bottom, polystyrene) was used to conduct an antibiofilm assay. Test bacteria were cultivated in Tryptic Soy Broth (TSB) at 37°C for 24 hours, and the culture was refreshed overnight in TSB+2% glucose solution up to an OD_600_ equal to 1 (i.e., log phase). Twofold serial dilutions of MℓAgNPs were used to determine 50% inhibition of microbial growth, i.e., the IC_50_. The distinct wells of the microtiter plates were filled with 190 μL of fresh culture media and 10 μL of silver nanoparticles of four different strengths. All assays were performed in triplicate, and the samples were incubated in the static position in an incubator (Memmert, Germany) at 37°C for 24 hours. After 24 hours, the components of the test plate were removed, and the wells were gently washed with PBS (pH 7.4) three times to remove the free-floating and nonadherent bacterial cells. After that, the bacterial cells in the wells of the microtiter plates were fixed with 99% methanol for 15 minutes, and the wells were then filled with 0.1% crystal violet (CV) stain for 5 minutes. Afterwards, the wells were washed with sterile distilled water to remove any remaining dye. Immediately after drying completely, 200 μL of ethanol (95%, v/v) was added to each well of the microtiter plates. The absorbance (OD) was measured at 620 nm in a microplate reader (Multiskan^™^ FC Microplate Photometer, Thermo Fisher Scientific, USA). The following equation was used to calculate the percentage inhibition of biofilm formation:

$$\text{\%}\;Biofilm\;inhibition=\frac{OD\;of\;untreated\;control-OD\;of\;Biofilm}{OD\;of\;untreated\;control}\times 100$$

The assays were performed in triplicate and calculate the mean ± standard deviation. The MICs of MℓAgNPs for antibiofilm potential were expressed as the IC_50_ [28].

### Statistical analysis

The data were examined statistically by SPSS (version 15.0) software using two-way (ANOVA) approaches with a 5% probability. The main and interaction effects of the independent variables were significant (*p* = 0.001). The least significant difference (LSD) test was used to compare the means of all the treatments, and the results were also significant.

## Results

### Synthesis of silver nanoparticles

**Fig 3:**
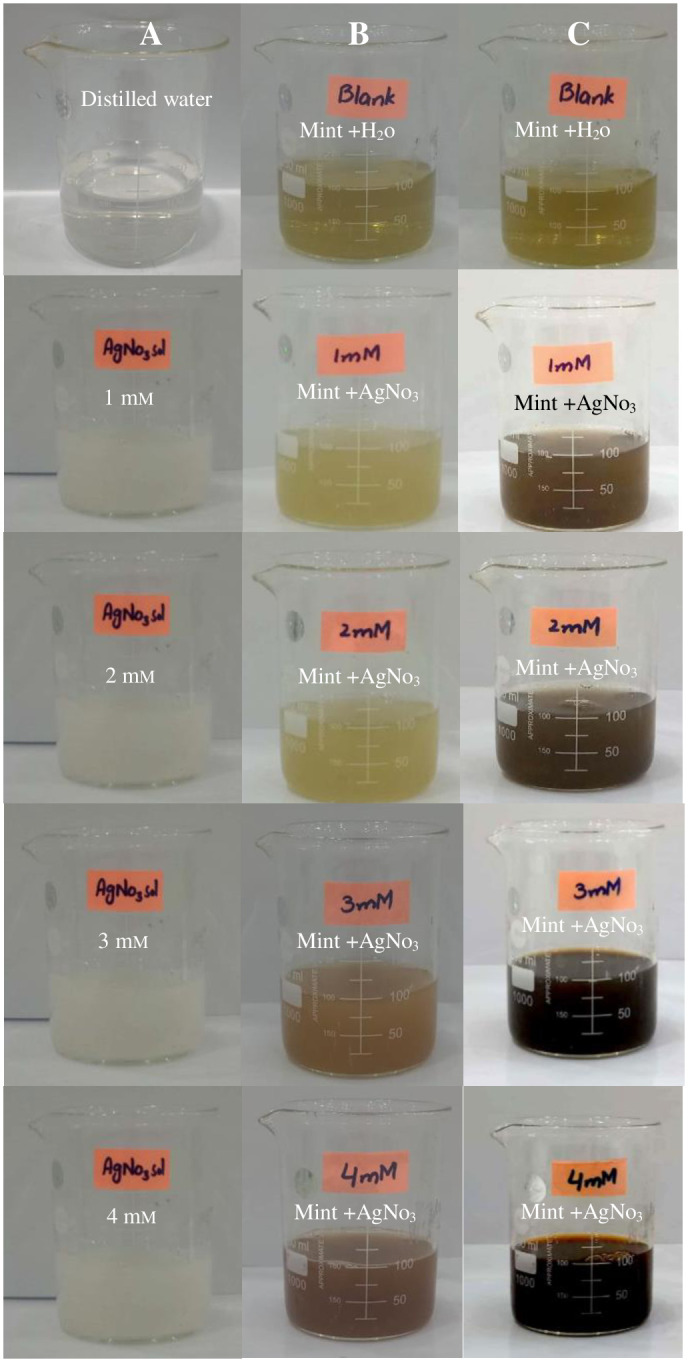
Color change of MℓAgNPs from different concentrations of silver nitrate solution. (A) Different concentrations of AgNO_3_. (B) Color of different concentrations at the time of preparation. (C) Color of reaction mixtures of different concentrations after 24 hours.

Four different concentrations of silver nitrate solutions were added to *M*. *longifolia* leaf extract, which were subsequently incubated overnight at room temperature to facilitate nanoparticle production. Successful formation of MℓAgNPs was confirmed by means of visual inspection of the color change (Fig 3), wherein the 1 mM and 2 mM reaction mixture converted to a light brown color and 3 mM and 4 mM concentrations turned into a deep brown color. With respect to the control mixture comprising only *M*. *longifolia* leaf extract and sterile, deionized water, no color change was detected, which is in accordance with the findings of Gurunathan *et al*., [29] and Sarkar and Paul [23]. The activation of surface plasmon resonance (SPR) in MℓAgNPs results in distinctive color changes during synthesis.

### Characterization of silver nanoparticles

#### UV visible spectroscopy

**Fig 4:**
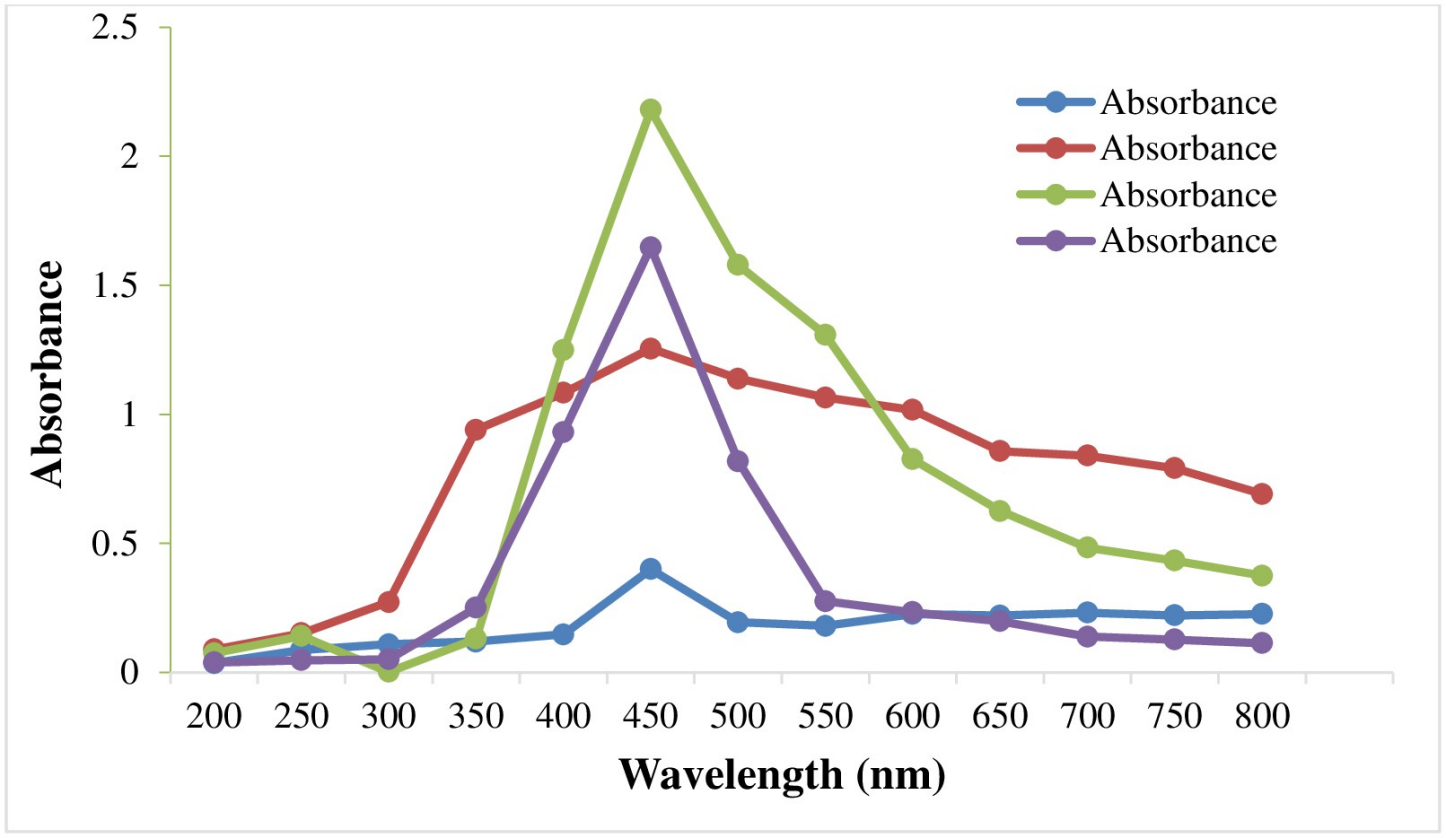
A UV‒Vis spectrophotometer was used to determine the presence of silver nanoparticles.

In the present study, the optimal concentration of MℓAgNPs was standardized by four different concentrations of silver nitrate solution. A spectrum in the visible region from 200 nm to 800 nm was obtained to demonstrate the presence of nanoparticles. The UV‒visible spectrum of the silver nanoparticles showed a strong and broad peak at 450 nm. The optimal silver nitrate concentration suitable for silver nanoparticle synthesis was found to be 4 mM (Fig 4), which was then selected and proceeded further.

#### Fourier Transform Infrared Spectroscopy (FTIR)

**Fig 5:**
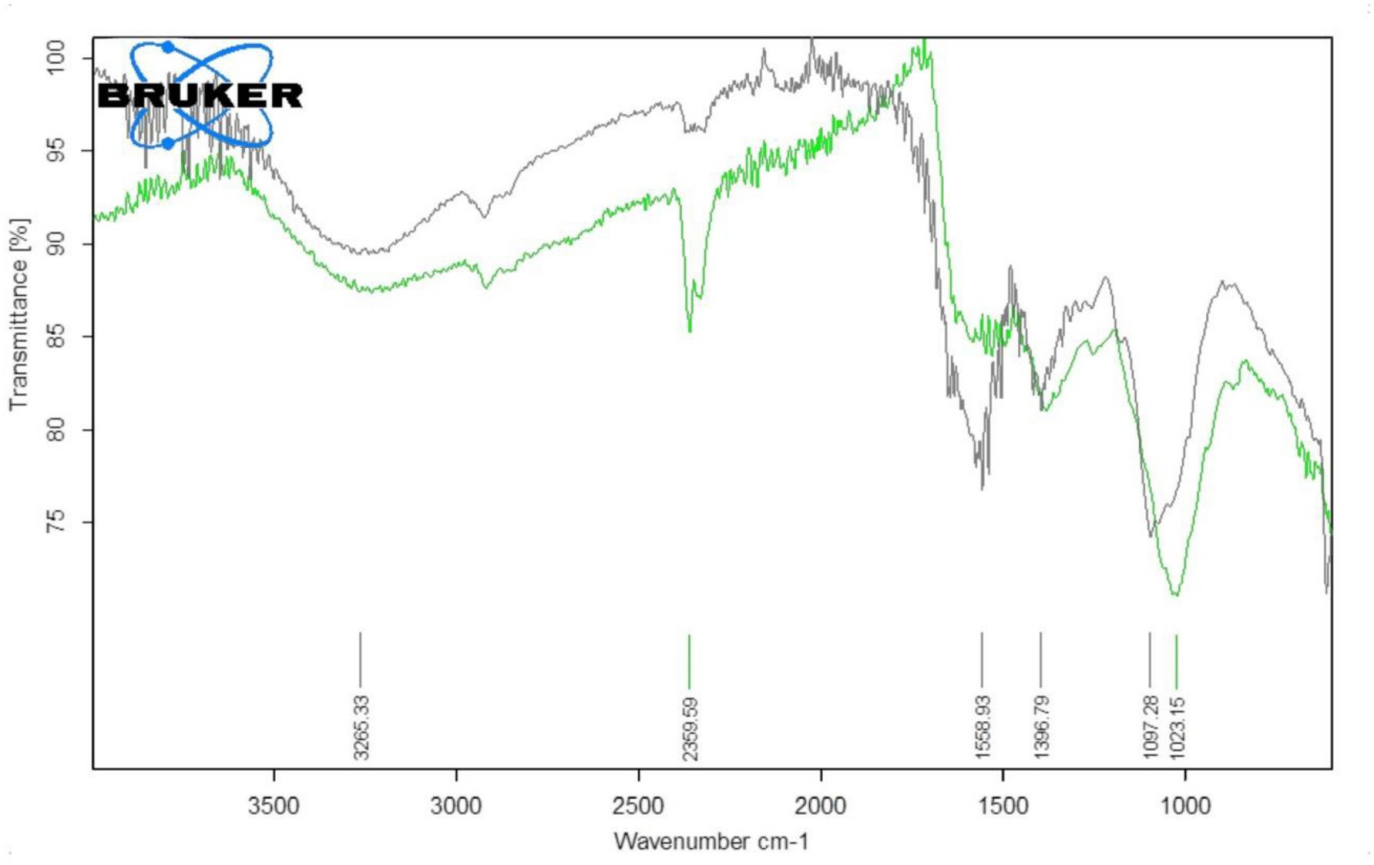
Fourier transform infrared (FTIR) spectra of MℓE (black) and MℓAgNPs (green).

FTIR analysis was used to characterize the surface chemistry of silver nanoparticles synthesized from *M*. *longifolia* leaf extract by using their 4mM concentration. The FTIR spectra was collected at 4 cm^-1^ resolution in transmission mode (3500–1000 cm^-1^). The spectra of *M*. *longifolia* leaf extract exhibited a peak at 3265.33 cm^-1^, which indicated the presence of O-H groups. The second peak at 1585.93 cm^-1^ was attributed to strong C = C stretching. Similarly, characteristic peaks at 1396.79 cm^-1^ and 1097.28 cm^-1^ indicated moderate C–N and C–O stretching, respectively, as shown in Table 1. The silver nanoparticle interactions with the chemical compounds found in the *M*. *longifolia* leaf extract revealed strong peaks at 2359.59 and 1023.15 cm^-1^. The highest absorption peak at 2359.59 cm^-1^ strongly indicates O-H stretching of C = O, and a distinguishing peak at 1023.15 cm^-1^ was detected as strong C-O stretching due to silver nanoparticle formation as shown in Fig 5 [30].

**Table 1:** The characteristic peaks of MℓAgNPs with their functional groups.

MℓE Peaks	MℓAgNPs Peaks	Functional Group	Compound Class
3265.33 cm^-1^		O-H	Hydroxyl alcohol and phenols
1585.93 cm^-1^		C = C	Alkene
1396.79 cm^-1^		C-N	Amine
1097.28 cm^-1^		C-O	Carbonyl group
	2359.59 cm^-1^	O-H	Carboxylic acid
	1023.15 cm^-1^	C = O	Carbonyl group

#### X-ray diffraction analysis (XRD)

**Fig 6:**
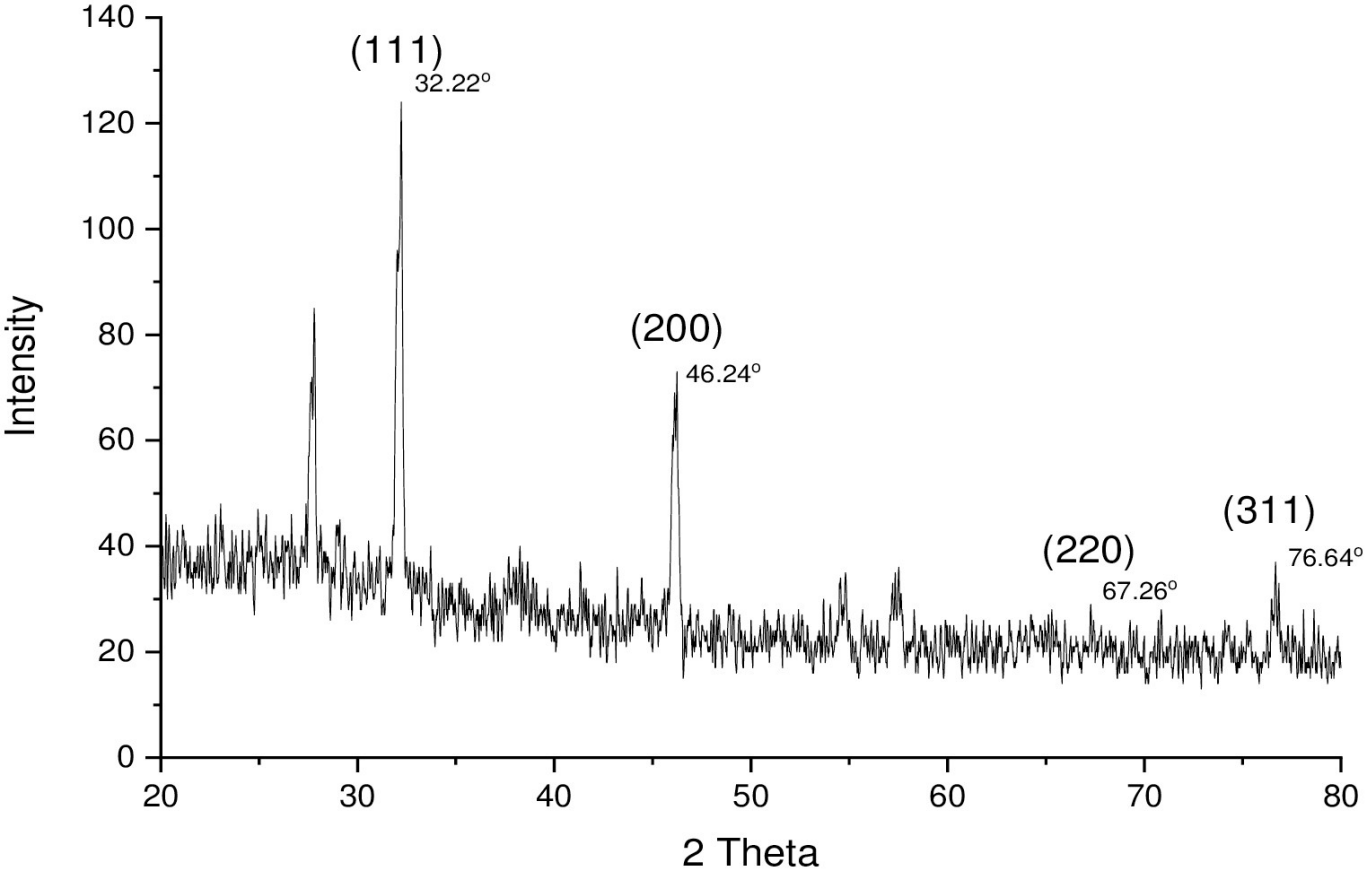
XRD pattern of MℓAgNPs.

The X-ray diffraction (XRD) pattern was taken by means of CuKα radiation with the help of a nickel monochromator (with JCPDS file number: 04-005-4584). The range was set at 2θ from 20° to 80°. The XRD patterns of the MℓAgNPs were characterized by four diffraction peaks at 32.22, 46.24, 67.26 and 76.64 cm^-1^, which indexed the (111), (200), (220), and (311) planes of the silver nanoparticles, respectively (Fig 6) [31].

#### Dynamic light scattering (DLS)

**Fig 7:**
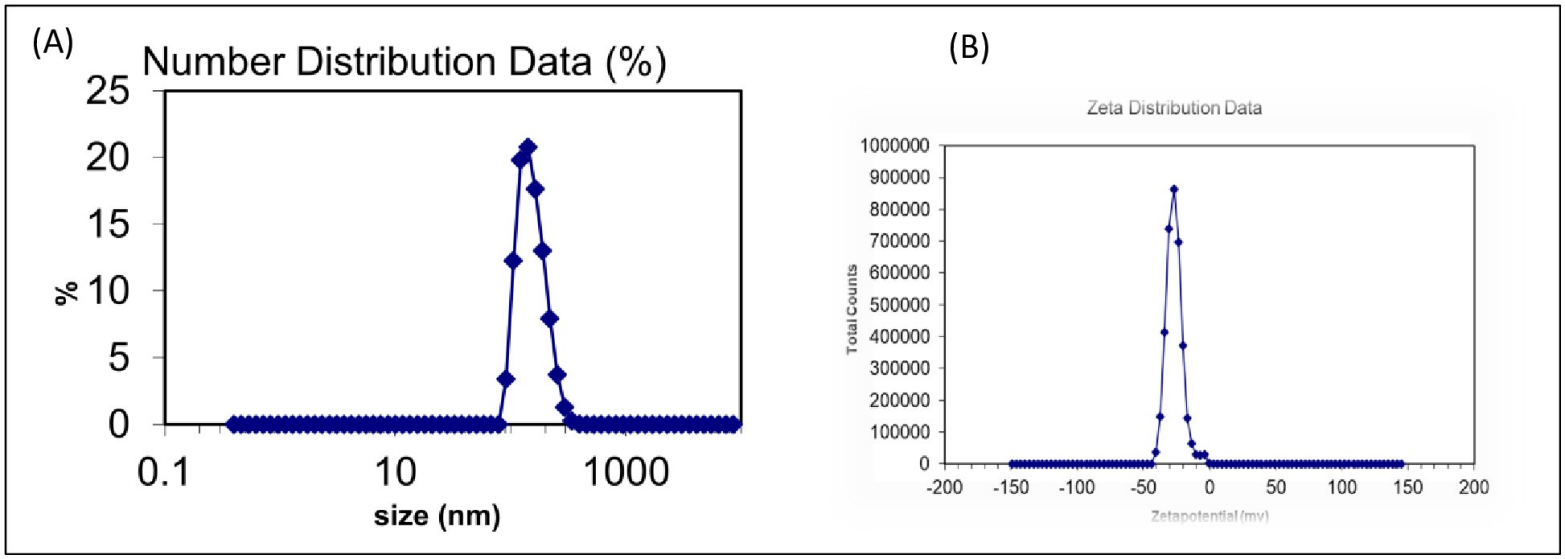
(A) Dynamic light scattering (DLS) and (B) zeta potential measurements.

The hydrodynamic size of the MℓAgNPs was analyzed at 25°C on a Zetasizer (Malvern, UK). The hydrodynamic size of the silver nanoparticles was 33.79 nm. A polydispersity index (PDI) of 0.462 demonstrated that the silver nanoparticles were monodispersed, as shown in Fig 7A [32].

#### Zeta potential measurements

The zeta potentials of the MℓAgNPs were found to be -26.7 mV and -5.45 mV. There were two peaks corresponding to silver nanoparticles, which indicated the presence of two major subpopulations of silver nanoparticles with different intensities. The peak with a lower intensity at -5.45 mV corresponded to smaller nanoparticles, and the peak with a higher intensity at -26.8 mV corresponded to larger silver nanoparticles, as shown in Fig 7B [32, 33].

#### Electron microscopy analysis

**Fig 8:**
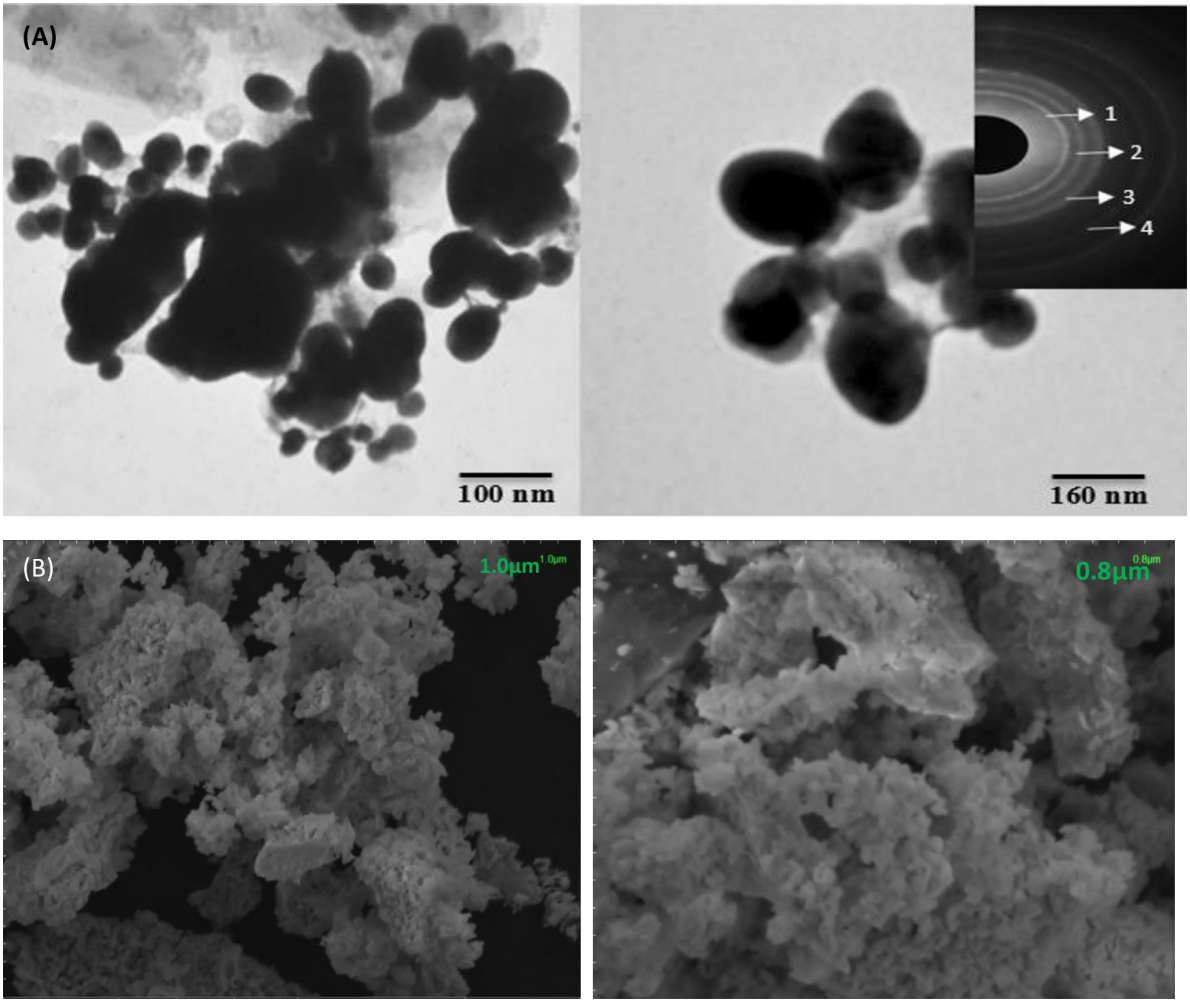
(A) TEM analysis of silver nanoparticles. (B) SEM analysis of silver nanoparticles.

The size of silver nanoparticles synthesized from *M*. *longifolia* leaf extract was examined by TEM analysis. The circular rings 1, 2, 3, and 4 are the result of reflections from the fcc silver lattice planes (111), (200), (220), and (311), respectively, as shown in Fig 8A. The interior circular diffraction pattern indicates that the silver nanoparticles are crystalline in nature. The size of the silver nanoparticles was found to be varied [34]. SEM was performed to determine the size and surface morphology of MℓAgNPs. SEM analysis indicated that the silver nanoparticles were uniform, spherical in shape, and distinct in nature, as shown in Fig 8B [28].

#### Antibacterial activity of silver nanoparticles

**Fig 9:**
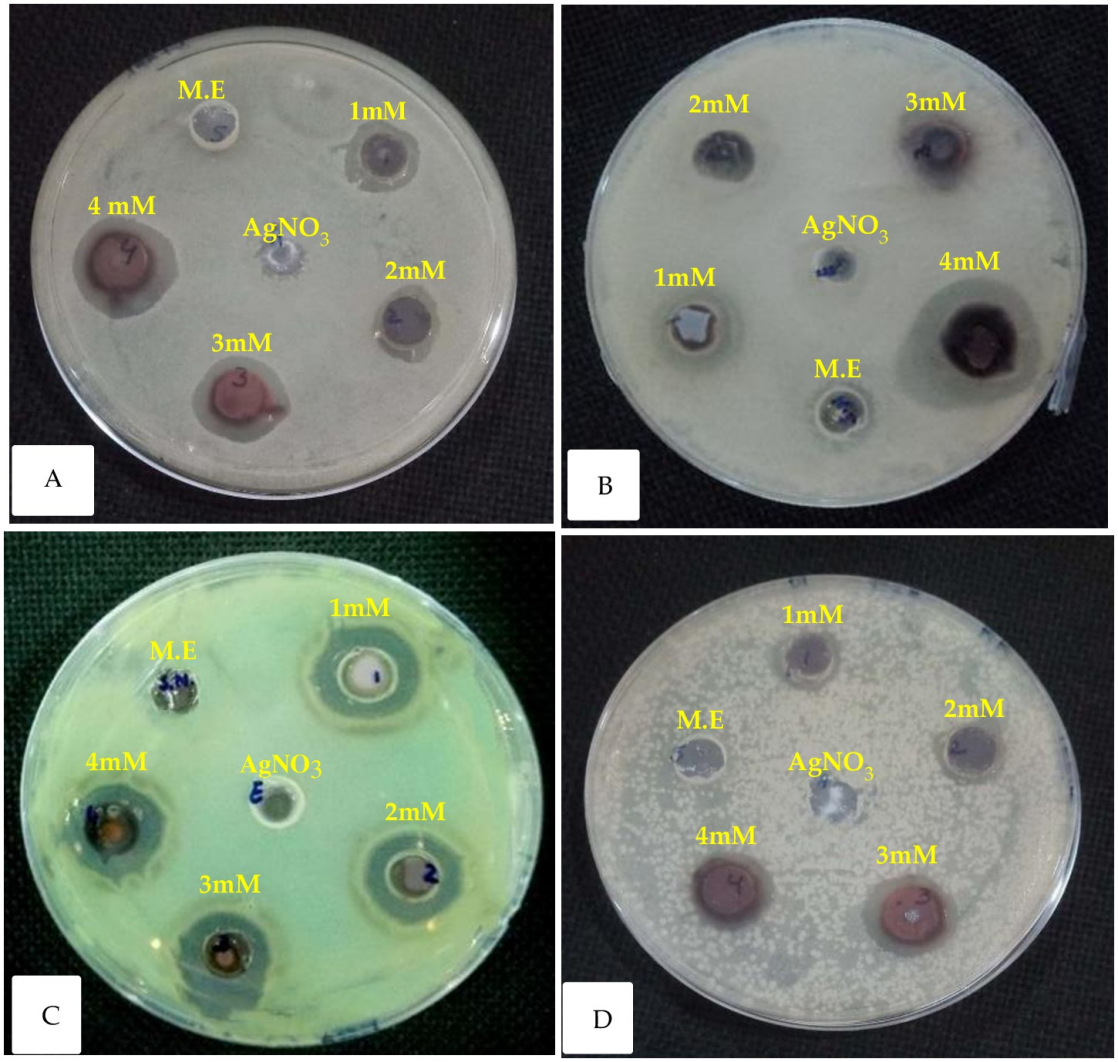
Antibacterial activity of four different concentrations of MℓAgNPs. Mint extract (M.E) and silver nitrate (AgNO_3_) were used as blanks for **(A)** *Escherichia coli*, **(B)** *Klebsiella pneumoniae*, **(C)** *Pseudomonas aeruginosa*, and **(D)** *Staphylococcus aureus*.

In the present research, the diameter of the inhibition zone increased to 20 mm with increasing concentrations of silver nitrate salt in the silver nanoparticles for each bacterial species (Table 2). The inhibition zone determines the antibacterial effect of silver nanoparticles. Furthermore, the MℓAgNPs exhibited notable inhibitory effects on *E*. *coli*, *K*. *pneumoniae*, *P*. *aeruginosa*, and *S*. *aureus*, as shown in Fig 9.

**Table 2:** Antibacterial activity of silver nanoparticles at different concentrations (zone of inhibition in mm) against four types of different pathogenic bacteria.

Bacterial species	Zones of inhibition (mm) Mean±SD					
	AgNo_3_ solution	*M*. *longifolia* leaves Extract	1 mM	2 mM	3 mM	4 mM
***E*. *coli***	_	9±0.28	13±0.34	15±0.15	16±0.34	18±0.14
***K*. *pneumonia***	_	10±0.25	15±0.15	16±0.26	18±0.54	20±0.25
***P*. *aeruginosa***	_	9±0.40	13±0.25	14±0.28	16±0.47	20±0.15
***S*. *aureus***	_	10±0.4	14±0.13	15±0.5	16±0.24	18±0.47

#### Determination of MIC and MBC

The values of minimum inhibitory concentration (IC_50_) of four different concentrations of MℓAgNPs against different bacterial pathogens are given in Table 3. After incubating for 24 hours at 37°C, turbidity was observed. The MIC increased by increasing the silver nitrate salt concentration in the silver nanoparticles. MℓAgNPs with a strength of 4 mM had potent antibacterial activity, with a very low value (IC_50_) of 0.03 μg/mL in *K*. *pneumoniae*. The MIC suspensions of all the bacterial strains were inoculated on nutrient agar plates and incubated at 37°C for 24 hours, after which no bacterial growth was observed. This result confirmed that MℓAgNPs at all concentrations have antibacterial activity.

**Table 3:** Minimum inhibitory concentrations (MICs) of MℓAgNPs at four different concentrations against four types of pathogenic bacteria.

Conc. of MℓAgNPs	*E*. *coli*		*K*. *pneumonia*		*P*. *aeruginosa*		*S*. *aureus*	
	MIC (μg/mL)	MBC (μg/mL)	MIC (μg/mL)	MBC (μg/mL)	MIC (μg/mL)	MBC (μg/mL)	MIC (μg/mL)	MBC (μg/mL)
**1 mM**	5±0.01	10±0.005	0.31±0.05	0.62±0.04	0.62±0.01	1.25±0.002	0.62±0.05	1.25±0.004
**2 mM**	2.5±0.02	5±0.002	0.15±0.01	0.31±0.09	0.31±0.02	0.62±0.001	0.62±0.07	1.25±0.005
**3 mM**	1.25±0.03	2.5±0.01	0.07±0.02	0.15±0.02	0.15±0.04	0.31±0.004	0.15±0.01	0.31±0.002
**4 mM**	0.62±0.01	1.25±0.001	0.03±0.01	0.078±0.01	0.07±0.02	0.15±0.001	0.07±0.02	0.15±0.001

#### Antibiofilm activity of silver nanoparticles

**Fig 10:**
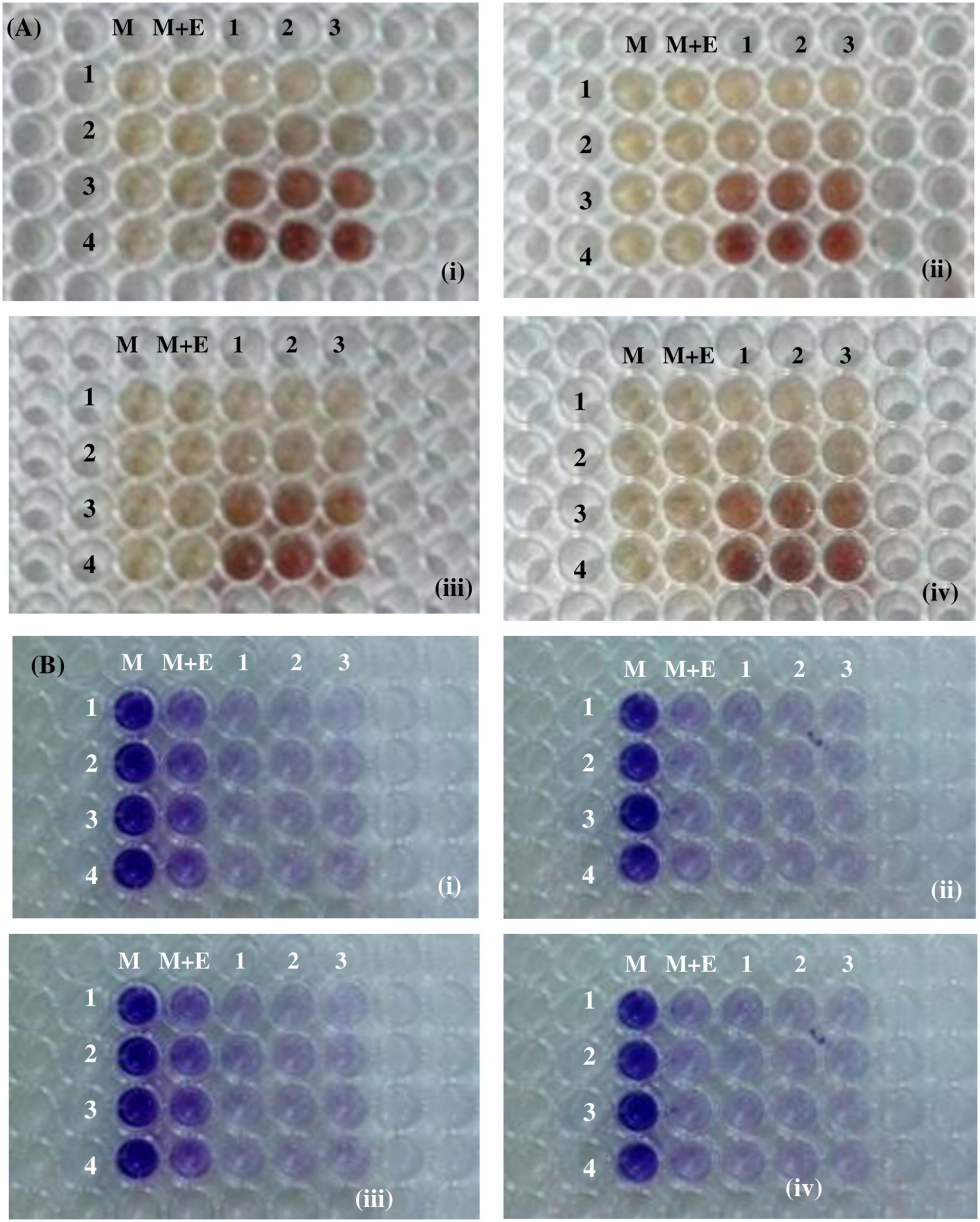
Antibiofilm activity of MℓAgNPs of four different strengths against four clinical strains. (A) Culture plate before staining after 24 hours of incubation, (B) after staining with 0.1% CV; (I) *E*. *coli*, (ii) *K*. *pneumoniae*, (iii) *P*. *aeruginosa*, and (iv) *S*. *aureus*. (Horizontal side) M = culture blank, M+E = Mint extract + culture as a control; 1, 2, and 3 are triplicates. (Left side) 1 to 4 are the concentrations (i.e., 1 mM to 4 mM of MℓAgNPs).

**Fig 11:**
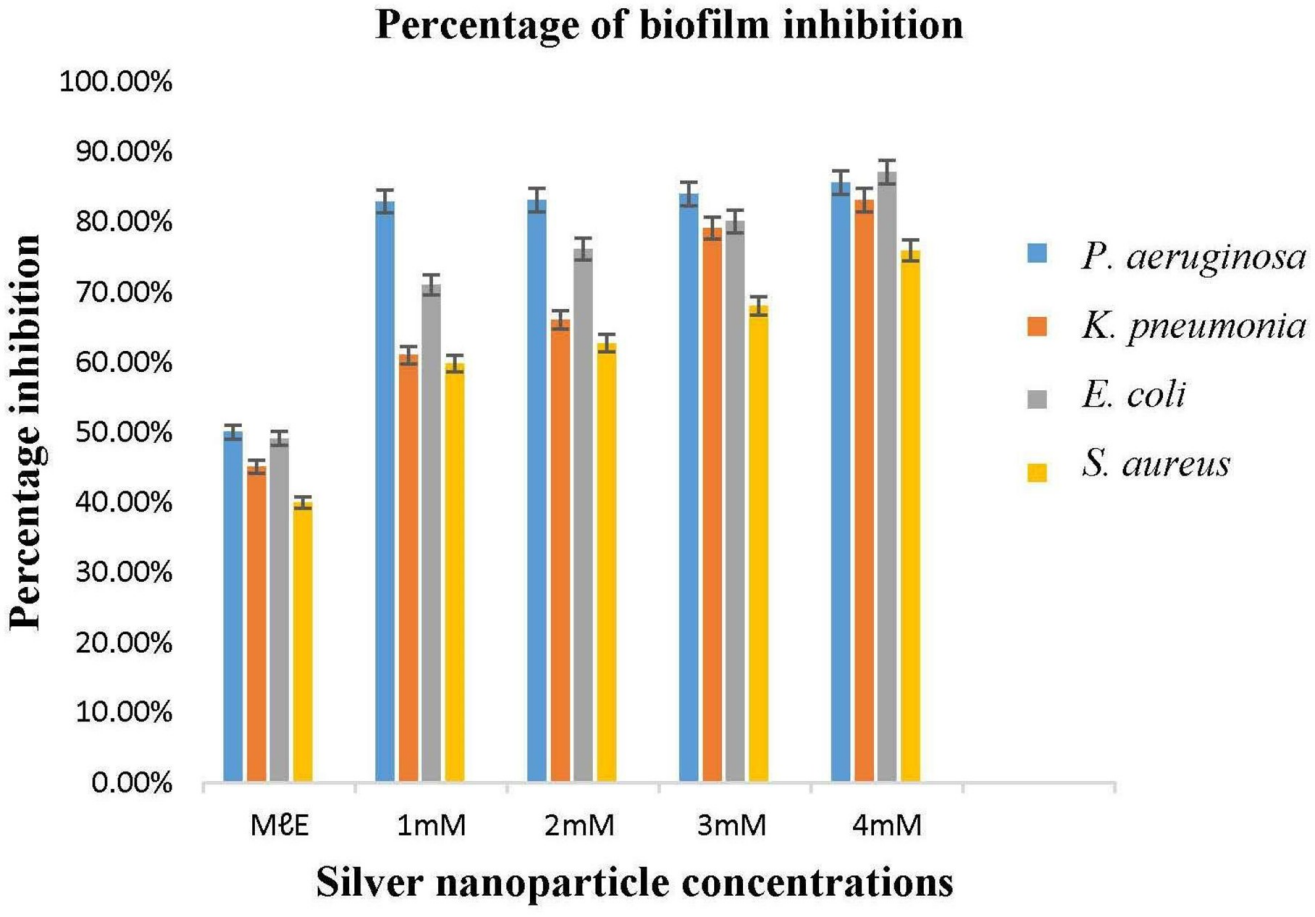
Graphical representation of the percentage inhibition of the antibiofilm assay, where p = 0.001 was considered to indicate statistical significance.

In current study, the dose-dependent antibiofilm effects of MℓAgNPs were assessed in vitro against biofilm-forming bacteria (i.e., *E*. *coli*, *K*. *pneumoniae*, *P*. *aeruginosa*, and *S*. *aureus*) (Table 4). Individual bacterial species were cultivated for 24 hours on 96-well microtiter plates before being treated with different strengths of synthesized MℓAgNPs at the respective IC_50_ values (Table 3). The results of the antibiofilm assay indicated that MℓAgNPs inhibited the biofilm formation of different bacterial species, especially in comparison with the positive and negative controls used in this study (Fig 10). In the case of *E*. *coli*, the IC_50_ (i.e., 5±0.01 μg/mL) of 1 mM MℓAgNPs exhibited 71% biofilm inhibition, whereas the IC_50_ (i.e., 0.62±0.01 μg/mL) of 4 mM MℓAgNPs demonstrated 80.09% biofilm inhibition. This increasing trend in biofilm inhibition with increasing strength of MℓAgNPs was also found for *K*. *pneumoniae*, *P*. *aeruginosa* and *S*. *aureus*. Overall, silver nanoparticles, regardless of the extract concentration used in their synthesis, had a great minimum inhibitory effect on biofilm formation [32, 33]. A graphical representation of the percentage inhibition of the antibiofilm assay is shown in Fig 11.

**Table 4:** Percentage inhibition values of the antibiofilm assay.

Biofilm	*M*. *longifolia* extract (control)	Strengths of MℓAgNPs			
		1 mM	2 mM	3 mM	4 mM
*E*. *coli*	49.09%	71%	76.12%	80.06%	87.09%
*K*. *pneumoniae*	45.07%	60.99%	66.02%	79.09%	83.11%
*P*. *aeruginosa*	50.00%	82.9%	83.06%	84.01%	85.60%
*S*. *aureus*	40.00%	59.78%	62.67%	68.01%	75.90%

## Discussion

The main goal of this research was to synthesize silver nanoparticles by reducing and stabilizing them with *M*. *longifolia* leaf extract. It seems like there was no observed color change in the control mixture containing only *M*. *longifolia* leaf extract and sterile, deionized water, that is in accordance to the findings of Gurunathan *et al*. [29] and Sarkar and Paul [23]. The authors stated that the activation of surface plasmon resonance (SPR) in silver nanoparticles can be due to distinctive color changes. Silver nanoparticles were studied using UV‒Vis spectroscopy, which is a well-established approach. The continuous synthesis of silver nanoparticles was monitored by UV‒Vis spectrophotometric analysis, and results were used to investigate the size- and shape-controlled nanoparticles in aqueous solutions. The absorbance peak at 450 nm is attributed to SPR of silver nanoparticles, that is established by surface plasmon stimulation of outer surface of silver nanoparticles and is triggered by an electromagnetic field in ultraviolet spectrophotometry that is according to the study of Awda *et al*. [8, 10].

The *M*. *longifolia* contained an abundance of chemical compounds, i.e., tannoids, monosaccharides, galactose, glucose, fructose, triterpene glycosides, terpenes, corticosteroids, coumarins, flavonoids, carbohydrates, and soluble starch. These compounds could have a role in reducing silver ions to metallic silver [35]. The FTIR spectra were used for the classification of functional groups of these chemical compounds using 4 mM concentration of silver nanoparticles, that are involved in production and stability of MℓAgNPs. In FTIR spectra the characteristic peak at 3265.33 cm^-1^ completely disappeared, demonstrating that the O-H group was convoluted during silver nanoparticles formation, and the peak observed at 2359.59 cm^-1^ indicated the formation of MℓAgNPs, as shown in Fig 5. Our findings are closely related with the results reported by Rauf *et al*. [8] as according to their study, the FTIR analysis indicated the presence of significant functional groups such as tannins, saponins, and phenolic flavonoids.

XRD analysis was performed to determine the crystalline nature of silver nanoparticles (4 mM conc.) synthesized from *M*. *longifolia* leaf extract. The XRD pattern was consistent with prior studies [1, 36]. The XRD results showed that the silver nanoparticles formed by the Ag+ ion reduction process and are crystalline in nature having no other impurities.

DLS analysis was performed to determine the hydrodynamic size of silver nanoparticles synthesized from *M*. *longifolia* leaf extract [37]. The hydrodynamic size of silver nanoparticles was 33.79 nm. The magnetic field density determines the metallic core size and surface coating of silver nanoparticles during Brownian motion. According to DLS analysis, silver nanoparticles are monodispersed in nature. Monodispersed silver nanoparticles contain one population of particles, and polydisperse silver nanoparticles contain more than one population of particles. Silver nanoparticles less than 100 nm in size have greater antimicrobial activity. Smaller nanoparticles are more easily incorporated into cells than larger nanoparticles. The number of cells is strongly dependent on the nanoparticle size [20, 32, 33].

The surface charge on silver nanoparticles was determined by ZP analysis. The 4 mM concentration of silver nanoparticles was used during the analysis. The magnitude of the zeta potential determines the potential stability of silver nanoparticles. Zeta potential greater than ±30 mV is considered to indicate a stable particle. A high surface electrical charge gives rise to an increased resistance against agglomeration, as stated by Sankar *et al*. [32]. The -5.45 mV and -26.7 mV values of silver nanoparticles indicated the stability of silver nanoparticles according to Bhattacharjee [33]. The surface negative charge of synthesized silver nanoparticles is due to stabilizing action of biomolecules present in the *M*. *longifolia* leaf extract, such as polyphenols, alkaloids, flavonoids, and tannins. The lower and higher intensity peaks at -5.45 mV and -26.7 mV corresponded to the smaller and the larger silver nanoparticles, respectively.

TEM analysis was performed to determine the size and internal morphology of silver nanoparticles (4 mM conc.) synthesized from *M*. *longifolia* leaf extract. TEM analysis revealed the formation of spherical silver nanoparticles with an internal morphology and a size ranging from 60–158 nm. In accordance with the study of Singh *et al*. [34], the TEM results showed that the silver nanoparticles are crystalline in nature.

SEM analysis was performed for the determination of size and surface morphology of MℓAgNPs (4 mM) synthesized from *M*. *longifolia* leaf extract. SEM analysis results are in accordance with Mohanta *et al*. [38] and indicated that the silver nanoparticles were uniform, spherical in shape with few variations and distinct in nature, as shown in Fig 8B.

The antibacterial activity results showed that plant-derived silver nanoparticles at four different concentrations can limit bacterial growth in vitro compared to that of non-treated bacteria or a negative control, and their activity increased with increasing concentrations of silver nitrate salt in preparation of silver nanoparticles. According to previous reports, nanoparticles are very harmful and toxic to pathogenic microorganisms. It has been demonstrated that silver nanoparticles have numerous antioxidant and antimicrobial properties hence they are useful as an antibacterial agent. The inhibitory mechanism of silver nanoparticles on microorganisms is not fully known yet. There are various possibilities, such as nanoparticles penetration inside the cell membrane, or interactions with phosphorus holding compounds such as DNA, obstruction of the replication process, and finally subsequent cell death of microorganisms [39]. It is also possible that a significant reaction between silver ions and the thiol group of essential enzymes inactivates them [40].

Moreover, in present research, MIC and MBC of MℓAgNPs at four different concentrations were evaluated. The values of MIC and MBC were significantly lower than those reported by Shaik *et al*. [41]. Lower MIC values indicate greater antimicrobial potential. In the present study, these significant differences in the MIC and MBC may have obtained because of the varying sizes of the silver nanoparticles.

Silver nanoparticles were also used to determine their antibiofilm effect against various strains of gram-positive and gram-negative bacteria that take part in biofilm formation [42, 43]. Different concentrations of silver nanoparticles inhibited biofilms to different extents compared to the control (*M*. *longifolia* extract). The 4 mM concentration of silver nanoparticles had the maximum percentage of biofilm inhibition among all four types of bacterial strains. In case of *P*. *aeruginosa*, 4 mM concentration of silver nanoparticles led to 85.60% biofilm inhibition. Similarly, the biofilm inhibition percentages for *E*. *coli*, *S*. *aureus*, and *K*. *pneumoniae* were 87.09%, 75.90%, and 83.11%, respectively, at the 4 mM concentration of silver nanoparticles. There are almost similar results in Mohanta *et al*. study [28]. Along with the correlation of silver nanoparticles to natural agents, the use of natural reducing agents found in plants as well as in microorganisms and microbial molecules developed by a microbe or plant are more profitable and safer for the biosynthesis of metal nanoparticles. In biomedical sciences, this line of research should be further investigated. Only a small amount of research has been performed on antibiofilm effect of silver nanoparticles [44]. In a study, Dashora *et al*. [45] used leaves extract of *Polyalthia longifolia* to synthesize silver nanoparticles and explored their antifungal activity against phytopathogen *Alternaria alternata*. Similarly, in another study, silver nanoparticles were synthesized from leaves extract of *Plantago ovata*, and their potential as antifungal agents and for removing organic dyes was evaluated [46]. AgNPs were found to be effective in inhibiting *A*. *alternata* fungal growth. Dhaka *et al*. [47] also prepared silver nanoparticles by lead extract of *Balanites aegyptiaca* to determine their antifungal and catalytic dye degradation potential. In another study, Rauf *et al*. [8] used a methanolic extract of *M*. *longifolia* to synthesize silver nanoparticles. Bacterial biofilms are formed as a result of bacterial cells synthesizing and secreting exopolysaccharides (EPSs), that are needed for biofilm development [48]. Bacteria respond to ecological stimuli that cause exopolysaccharides to be produced. As a consequence, if exopolysaccharide formation can be slowed or inhibited, biofilm formation can also be limited. This present research study based on the concept of antibiofilm effect of silver nanoparticles [49]. High levels of drug-resistant pathogens have recently been developed in semitropical environments and pose major health problems [50, 51]. The recent use of antibiotics for combating pathogens has led to rapid inadequate control of novel strains of current diseases causing pathogenic agents and new living organisms. Curated antimicrobial agents with high-performance nanotechnology introduce an innovative approach for the treatment of microbial pathogens that are resistant to current medications and for biofilm treatment. The present research indicates the potential of using plant-derived silver nanoparticles to inhibit biofilm formation in a new way to effectively treat a number of diseases caused by pathogenic microbes owing to their ability to produce biofilms [52, 53].

## Conclusions

In the present study, green chemistry was used for the synthesis of silver nanoparticles from the leaves of a native variety of *Mentha longifolia*, which is an eco-friendly, cost effective, and quick approach. The different strengths of the silver nanoparticles were optimized and evaluated for their ability to inhibit biofilm formation in comparison to that of the control (*M*. *longifolia* extract). A 4 mM concentration of silver nanoparticles had the maximum percentage of biofilm inhibition for all four types of bacteria. With respect to the *P*. *aeruginosa*, silver nanoparticles of 4 mM concentration led to 85.60% biofilm inhibition. Similarly, 4 mM concentrations of *E*. *coli*, *S*. *aureus*, and *K*. *pneumoniae* silver nanoparticles inhibited up to 87.09%, 75.90%, and 83.11%, respectively. For our best knowledge, it is the first study on the use of the green synthesized silver nanoparticle MℓAgNP as an antibiofilm agent. The extract of *M*. *longifolia* has been identified as having excellent commercial potential for the synthesis of silver nanoparticles. Plant leave-derived silver nanoparticle MℓAgNPs presented substantial antibacterial and antibiofilm effects on various human pathogens of clinical importance. In comparison to the physical or chemical synthesis of silver nanoparticles, the use of plant extract has an advantage due to their ability to stabilize silver nanoparticles, their own high antimicrobial activities, greater effectiveness, and minimal toxic effects. Their auspicious application constitutes an environmentally pleasing strategy for silver nanoparticle synthesis.
